# How Do COVID-19 Risk, Life-Safety Risk, Job Insecurity, and Work–Family Conflict Affect Miner Performance? Health-Anxiety and Job-Anxiety Perspectives

**DOI:** 10.3390/ijerph20065138

**Published:** 2023-03-14

**Authors:** Wei Zhang, Dongxiao Gu, Yuguang Xie, Aida Khakimova, Oleg Zolotarev

**Affiliations:** 1 School of Management, Hefei University of Technology, Hefei 230009, China; 2 Scientific-Research Center for Physical-Technical Informatics, Russian New University, Moscow 105005, Russia

**Keywords:** miners, perception of COVID-19 risk, life-safety risk, perceived job insecurity, work–family conflict, anxiety, job performance

## Abstract

The coronavirus pandemic (COVID-19) has created challenging working conditions in coal-production activities. In addition to the massive loss of resources for miners, it has had a devastating impact on these individuals’ mental health. Based on the conservation of resources (COR) theory and a resource-loss perspective, this study examined the impact of COVID-19 risk, life-safety risk, perceived job insecurity, and work–family conflict on miners’ job performance. Moreover, this study investigated the mediating role of job anxiety (JA) and health anxiety (HA). The study data were collected through online structured questionnaires disseminated to 629 employees working in a coal mine in China. The data analysis and hypothesis generation were conducted using the structural equation modeling (partial least squares) method. The results demonstrated that the perception of COVID-19 risk, life-safety risk, job insecurity, and work–family conflict negatively and significantly impacted miners’ job performance. In addition, JA and HA negatively mediated the relationships between the perception of COVID-19 risk, life-safety risk, perceived job insecurity, work–family conflict, and job performance. The findings of this study can give coal-mining companies and their staff useful insights into how to minimize the pandemic’s effects on their operations.

## 1. Introduction

Since December 2019, the COVID-19 pandemic has heavily influenced the lives and health of people worldwide, thus posing a significant challenge to industrial production [1]. The continued mutation and rapid spread of the virus have threatened people’s health worldwide and accelerated the population’s mortality rate [2]. The crisis has been long-lasting and widespread, causing significant psychological problems in all professions [3]. Significantly, the emergence of a significant risk of viral infection with effects on psychological well-being has taken a tremendous toll on the energy-extraction industry, especially coal mining.

Coal is an important energy source and industrial raw material. Furthermore, coal mining is a special energy-production industry, which strongly supports the steady and rapid development of China’s economy and society. As of 2022, China had 4600 coal mines, in which more than 2.4 million people were employed. Miners must travel hundreds to thousands of meters underground to work in harsh conditions [4]. Occupational and equipment factors cause various occupational injuries to miners, such as respiratory diseases, bone diseases, and heart diseases, which not only cause physical pain to miners but also severely affect their psychological health [5]. To prevent the spread of the virus, many coal mines have taken several measures, such as closed management and non-stop production, to ensure the supply of coal in a stable and orderly manner. As a result, many miners have been required to work and live in closed areas of mines, which creates many challenges for their psychological well-being and family lives.

Miners are among the most hazardous occupations [6]. In previous studies of miners, an average of 25.5% of miners from 1029 miner households in four regions of Ghana were injured in one year. Miners still have high injury rates despite regulations, automation, and safe production practices to reduce workplace risks [7]. During the COVID-19 pandemic, as a typical labor-intensive industry, the coal industry is characterized by high personnel exposure and confined working environments, which makes the pressure on coal companies to prevent epidemics increase, and the coal-production volume is easily affected [8]. As the number of COVID-19 infections continues to rise, lockdowns and social quarantines have been imposed in mines to stop the spread of the virus [9]. Although proven to be a very effective method of physical protection, these have caused operational disruptions, lost production, and financial losses to coal companies. When COVID-19 was properly mitigated, miners returned to work [10]. However, they are still at high risk of infection in the face of an emergency outbreak [11]. When they or their close contacts are infected, they must again be forced into isolation, dramatically affecting their mental health and job performance.

Previous studies have shown that uncertain environmental factors (health crises, economic recessions, technological change, political insecurity, etc.) may lead to an increase in business shutdowns and disruptions, a decline in the workforce, an increase in social unemployment, and insecurity among employees about their jobs [12]. Therefore, COVID-19 can lead to more significant financial pressure on companies. Financial stress can create employee instability, such as job loss and salary reduction. These factors result in reduced or interrupted income (i.e., loss of financial resources) and decreased quality of life for employees [13].

Miners have long work cycles, which reduce the time spent with their families. In a related study, it was found that there was a strong correlation between miners’ anxiety disorders and work–family conflict [14]. Considering the poor working environment in coal mines, miners work under much higher stress than other occupational groups. These work-related factors can easily lead to work–family conflict [15]. Miners’ time with their families is further reduced, which makes them even less able to shoulder their family responsibilities during the COVID-19 pandemic [16].

Researchers generally agree that miners lack the resources to cope with risks and psychological problems effectively [17]. Based on the conservation of resources (COR) theory, job performance in the coal industry is largely influenced by the resources they possess. Therefore, miners are vulnerable to internal- and external-resource losses during crisis events, such as that brought about by COVID-19 [18]. The risks posed by COVID-19 result in a significant loss of resources (i.e., health, working conditions, finances, and family), which may affect miners’ mental health [5]. Various adverse psychological factors also tend to increase anxiety caused by occupational and physical stress, ultimately affecting miners’ productive performance [19]. Therefore, miners’ anxiety levels and job performance must be examined. This will contribute to the sustainable development of coal-mining companies and the stability of China’s energy economy.

In the previous literature on miners, many studies examined the effects of numerous occupational characteristics on the psychological issues and job performance of miners [4,5,6,7]. In contrast, few studies have been conducted on miners in the context of public-health-crisis events. As COVID-19 caused a gradual increase in the number of patients with anxiety, domestic and international scholars conducted empirical studies on different populations, but the studies on miners’ anxiety were insufficient. Furthermore, no COVID-19-related studies have examined miners’ anxiety and job performance from the perspective of resource loss. This paper attempts to fill this gap by expanding the literature on miners by exploring the impact of global public-health events on the coal industry.

Fundamentally, this study aims to provide a substantiated view of the adverse effects faced by miners during the pandemic crisis. This article illustrates the importance of challenging working conditions and maintaining employees’ behavioral and psychological resources. Furthermore, in order to address the negative impact of COVID-19 on miners, this paper investigates the relationship between resource loss and the job performance of miners based on COR. According to COR, COVID-19 increases the risk of virus infection and job insecurity in the surrounding environments of miners. The threat posed by COVID-19 causes miners to lose their health resources. The financial loss of society reduces the incomes of miners. The occupational risks and family–work conflicts caused by the specific nature of miners’ jobs can also increase miners’ anxiety levels. Therefore, this study first addresses the effects of COVID-19 risk, life-safety risk, perceived job insecurity, and work–family conflicts on job performance from a resource-loss perspective during the COVID-19 pandemic. Next, it explores whether employee anxiety mediates the effects of resource loss and job performance. Finally, it explores the impact of the degree of loss of resource elements on miners’ job anxiety and health anxiety. In summary, this study aims to assess the extent of miners’ perceived risk of the widespread COVID-19 and to examine the scientific literature on work–family conflict and occupational safety related to individual job performance.

The paper has the following structure. The issue is briefly introduced in the first section. The theoretical background and hypothesis development are presented in Section 2. In Section 3, the research methodology is highlighted, and the materials and methods used in this paper are introduced. Furthermore, the results of the investigation are presented in Section 4. The research discussion and conclusion are each illustrated in Section 5 and Section 6, respectively.

## 2. Theoretical Background and Hypothesis Development

### 2.1. The Conservation of Resources Theory

The COR theory is often used to discuss the adjustment and adaptation of individual-resource supply-and-demand situations. The theory holds that individuals will take actions to acquire, preserve, protect, and cultivate their valuable resources to balance the supply of and demand for these resources, which is referred to as resource acquisition. By contrast, the loss of resources is a crucial element that constitutes a stress-response mechanism. Individuals feel psychologically uncomfortable after investing resources and not receiving returns when suffering a potential or actual loss of resources [20]. Individuals feel less stress if they are able to preserve existing resources and replenish new resources properly. Hobfoll defined resources as materials (e.g., job compensation), conditions (e.g., marriage and employment), personal characteristics (e.g., personality traits), and energy (e.g., feelings of recognition and accomplishment) [18,21].

Stress occurs in individuals when their current resources are in danger of being lost or invested without gaining any future returns. Individuals respond with part of their resources when faced with stress situations. The negative effects of stress become apparent when individuals feel a lack of resources in situations in which resources are constantly lost but not replenished. People are at a high risk of infection due to the COVID-19 pandemic, which results in a loss of health resources. The COVID-19 pandemic has resulted in the loss of resources for working conditions, which threatens the lives of miners. Economic crises lead to job insecurity (e.g., job losses, pay cuts), causing a loss of financial resources. The deepening of work–family conflicts, which leads to the loss of condition resources (e.g., the loss of family-relationship resources). Thus, miners face losses of health, job, financial, and family resources as a result of the risks and challenges imposed by the pandemic crisis, which causes heightened stress (i.e., anxiety). When resource losses cannot be effectively compensated, negative attitudes and behaviors at work are adopted to protect available resources and reduce losses.

Given that resources are also used to prevent resource loss, stored resources are used to offset the pain caused by resource loss in relieving stress. Sustained pressure can lead to a rapid and consequential spiral of resource loss [22]. The COR theory is considered an alternative to assessment-based-stress theory [20]. This theory has been used successfully to predict stress in organizational and health settings, post-traumatic stress, and various other outcomes related to everyday stressors. Therefore, this paper studies the impact of resource loss caused by COVID-19 on anxiety and work performance based on the conservation of resource (COR) theory, which will help to provide insights into the mechanism of miners’ work performance.

### 2.2. Hypothesis Development

#### 2.2.1. Perception of COVID-19 Risk

The perception of COVID-19 risk refers to an individual’s awareness of the risk of infection with COVID-19 in the external environment, which can trigger perceptions of fear, stress, and risk in society. The disease, COVID-19, is an acute respiratory infection caused by a novel coronavirus that is widely spread and highly contagious [23]. These characteristics create significant psychological shock. The COVID-19 pandemic has created anxiety among the public. This scenario further increases the public uncertainty about the epidemic due to the lack of effective drugs for the treatment of COVID-19 and knowledge about this topic among the public [24]. Thus, the COVID-19 pandemic has significantly affected physical and psychological health [25].

Notably, many occupations offer a work-at-home setup [26]. However, miners can only complete their tasks at the mine shaft. Miners face health and safety risks associated with a high-risk workplace, making them vulnerable to serious viral infections [27]. The high mortality risk associated with COVID-19 can amplify personal anxiety, including job anxiety and health anxiety [28]. Recent studies have shown that COVID-19 causes severe mental health problems and significantly and positively affects the anxiety of frontline hotel employees [12]. Yuan et al. [29] confirmed that COVID-19 caused severe psychosomatic disorders (e.g., depression) among frontline workers in China. Kurceret al. [30] concluded that the fear due to COVID-19 can trigger cyber-hypochondria in students, leading to a disordered life and damaged health.

The COVID-19 pandemic significantly affects the country’s economic development, which first reduces employees’ job performance and, consequently, causes them occupational stress. Documentation has shown that anxiety generated by the COVID-19 pandemic is a common problem affecting employee performance [6]. The hazards associated with COVID-19 make people less focused on tasks linked to work, dramatically lowering their overall job performance [31]. Moreover, the COVID-19 epidemic disrupts regular operations and may increase people’s workloads and burdens at work, which lowers job performance [32]. Therefore, the following hypotheses were constructed:

**H1a.**  *Perception of COVID-19 risk has a positive impact on job anxiety.*

**H1b.**  *Perception of COVID-19 risk has a positive impact on health anxiety.*

**H1c.**  *Perception of COVID-19 risk has a negative impact on job performance.*

#### 2.2.2. Life-Safety Risk

Mining work is one of the most dangerous jobs in the world, and it is characterized by various occupational features, such as high loads, irregular life patterns, and work–life interference [33]. As a particular occupational group, miners perform several tasks, including lifting, bending, and standing in confined spaces, which threaten their safety and health.

Undoubtedly, miners work in physical environments with dust exposure, high temperatures, noise, and vibration [4]. These cause many occupational diseases among miners, such as respiratory diseases, musculoskeletal disorders, and hypertension. These occupational diseases may cause painful experiences, affect miners’ health, and increase negative emotions [34]. The personal, occupational, and equipment-related occupational risks that miners face may also contribute to their job anxiety [8]. One study found that poorer psychological well-being may arise for younger Western Australian miners (fly-in and fly-out) who are under travel quarantine and experience at least two COVID-19-related symptoms [35].

In addition, the hazardous nature of miners’ occupations reduces their health status and affects safe production in the mining industry. Kunda et al. [7] found that 265 miners (25.8% of all the miners surveyed) had moderate or severe absenteeism due to occupational hazards in the previous year. Widanarko et al. [36] confirmed that occupational diseases with lower-back symptoms caused miners’ absenteeism to grow and reduce the productivity of individuals. Life-safety risks (i.e., occupational risks) in the workplace may impair several aspects, such as individual health or job performance [37]. According to the COR theory, when an individual’s resources are lost and fail to produce the expected return after a large amount of resource investment, the anxiety and job performance generated by work stress increase. In summary, miners’ health resources are lost when prolonged underground production operations without air circulation damage their lungs. During the COVID-19 pandemic, the confined and small spaces underground was highly conducive to the spread of the virus. Infection with COVID-19 causes miners to become more anxious, which makes it difficult for them to work. Delays to miners’ work schedules affect their overall job performance [38]. Therefore, the following hypotheses were formulated:

**H2a.**  *Life-safety risk due to COVID-19 has a positive impact on job anxiety.*

**H2b.**  *Life-safety risk due to COVID-19 has a positive impact on health anxiety.*

**H2c.**  *Life-safety risk due to COVID-19 has a negative impact on job performance.*

#### 2.2.3. Perceived Job Insecurity

Hellgren observed that job insecurity is subjective, as employees hold uncertainty about the future, including doubts about employment [39]. Faced with the pandemic crisis, many industries underwent layoffs, resulting in significant job losses [13].

Employees’ job insecurity may change positively or negatively over time. Because of the urgent need for large numbers of healthcare workers to fight the outbreak of COVID-19, healthcare workers’ basic skills may not have been affected, making them likely to feel more secure [32]. In contrast, employees’ job insecurity threatens their status and prospects within their organization and can generate negative emotions [40]. Probst and Bazzoli [41] demonstrated that job insecurity causes poor work attitudes among workers and affects their safety-compliance behaviors. Aguiar-Quintana [42] demonstrated that job insecurity among hotel employees positively and significantly affects their psychological well-being (i.e., anxiety).

Job insecurity has a detrimental effect on job performance indirectly through anxiety. When employees’ psychological resources are excessively depleted, significant stress is generated, which leads to negative attitudes toward work and the organization. Guo et al. verified that job insecurity directly and significantly negatively affects highway drivers’ sense of life safety, based on the COR theory [43]. Jung demonstrated that job insecurity among hotel employees had a significant impact on their engagement with their jobs and desire to quit [44]. During the COVID-19 pandemic, normal production was heavily affected because of the phased spread of the epidemic. The surge of those infected with COVID-19 resulted in many miners being absent from work and low coal-mine attendance. This forced mine plants to take measures to shut down or reduce production, which may have affected miners’ job security (i.e., through the loss of economic resources). Given that job insecurity depletes an individual’s essential resources, it can take a significant psychological toll on employees, hinder creativity, and lead to rigidity in the workplace.

When the COVID-19 pandemic was at its most intense, coal mines took emergency measures, such as the closed management of mine areas, which prevented some employees from returning to work on time, causing job anxiety among miners. The continuous quarantine in the closed sites exposed employees to job insecurity, leading to low production [10]. Therefore, the following hypotheses were constructed:

**H3a.**  *Perceived job insecurity due to COVID-19 has a positive impact on job anxiety.*

**H3b.**  *Perceived job insecurity due to COVID-19 has a positive impact on health anxiety.*

**H3c.**  *Perceived job insecurity due to COVID-19 has a negative impact on job performance.*

#### 2.2.4. Work–Family Conflicts

Complex issues arise when employees experience stressful situations in terms of work and family, which leads to work–family conflicts [45]. For example, individuals cannot take on family responsibilities, such as picking up and dropping off children on time due to work commitments; furthermore, they cannot concentrate on work due to concerns about family members, resulting in inefficiency, etc. When individuals take on additional work roles, their responsibilities to their families are reduced. Individuals have limited time and energy; hence, competition between work and family for resources can occur frequently. The conflict between work and family can have various consequences, such as depression, physical and mental exhaustion, and increased mental stress, which can seriously affect individual’s physical and psychological health [46]. For companies, work–family conflict is detrimental to business performance and long-term development. During the COVID-19 pandemic, miners may have initially experienced home isolation, making them unable to return to their mine plants; subsequently, longer working hours may have made it difficult for miners to balance family and work, eventually leading to work–family conflict [16].

Occupational factors, such as continuous night work, poor working environments, and high work stress, among miners, can easily lead to work–family conflict. This conflict is associated with employee mental health and positively affects employees’ mood, anxiety, and psychiatric disorders [47]. In one study, work–family conflict was found to have a substantial positive impact on the depressive symptoms of doctors during COVID-19 [48]. Meanwhile, Panatik et al. [49] found that work–family conflict had a negative impact on teachers’ mental health.

Miners’ high-risk work environments and the intensity of their work tasks require them to be highly focused. When work–family conflicts occur, miners must spend significant time and energy adjusting their emotions and states, making it difficult for them to focus entirely on their work [50]. Zhang et al. [51] found that work–family conflict had a significantly negative effect on the job performance of subway employees during the COVID-19 pandemic. Bojan showed the indirect effect of work–family conflict with psychological safety as an intermediate variable in employees job performance [52]. On one hand, the isolation of some miners at home led to a lack of financial income, which intensified work–family conflicts. On the other hand, the increased labor intensity of workers on the job caused difficulties in balancing work and family time. All these factors led to anxiety among employees [53]. With the uncertainty and suddenness of coal-mine closures, work–family conflict has become a potential cause of miners withdrawing from their work and displaying a decline in performance [54]. Therefore, the following hypotheses are formulated:

**H4a.**  *Work–family conflicts have a positive impact on job anxiety.*

**H4b.**  *Work–family conflicts have a positive impact on health anxiety.*

**H4c.**  *Work–family conflicts have a negative impact on job performance.*

#### 2.2.5. Mediating Role of Job Anxiety

The anxiety that employees feel due to their workplace or work tasks is called job anxiety [55]. Job anxiety is manifested through employees’ nervousness about the functioning of their organization and their ability to meet the expectations of their employers [56].

Job anxiety, as a psychosocial risk factor, affects employees’ performance [57]. Because of COVID-19, the regular work schedules of miners have changed, which has had a substantial effect on their mental health. To prevent the spread of viruses, maintaining social distancing was a standard public-health prevention policy. During the COVID-19 pandemic, some coal mines have closed management policies, and miners live and work in the mines. The conditions forced miners out of their normal rhythms of life and triggered job anxiety [58]. Some studies have shown that the stigma toward COVID-19 (concerns about disclosure, public attitudes, and negative experiences) affects frontline health workers’ anxiety and job performance indirectly [59]. Given the uncertainty created by the COVID-19 pandemic, employee anxiety has devastated the changing work environment, resulting in a decrease in job performance.

Ensuring workplace safety is the core concern of miners [60]. Employees always worry about their physical condition while at work due to the harmful impacts of COVID-19 [61]. This work-related psychological worry affects employees’ expectations and, thus, reduces their performance [62].

The high risk associated with COVID-19 makes coal-mining schedules unstable, and the resulting corporate benefits expose employees’ families to financial hardship [63]. Job insecurity leads to negative employee attitudes toward work and reduces individual performance [64]. In terms of resource conservation, job insecurity depletes emotional resources and has an impact on employee behavior [65]. Miners experiencing job insecurity consume their emotional resources and, thus, refuse to comply with corporate rules and participate in productive activities [66].

The COVID-19 pandemic has an impact not only on miners’ work but also on their family lives. Work–family conflicts leave miners prone to psychological problems and may lead to coal-mine accidents [15]. During the COVID-19 pandemic, employees forced into isolation were prone to job worries and mental disorders, creating work–family conflicts and, ultimately, affecting employees’ organizational performance [67].

According to the COR theory, conserving resources when faced with adverse workplace situations has an incentive [17]. The presence of job anxiety directs employees’ energy resources to negative activities, such as worry and distress, rather than contributing based on productive behavior [56]. Therefore, the following hypotheses were constructed:

**H5.**  *Job anxiety negatively mediates the relation between resource loss (perception of COVID-19 risk, life-safety risk, job insecurity, work–family conflict), and job performance.*

#### 2.2.6. Mediating Role of Health Anxiety

Health anxiety is an irritation that occurs when employees are overly worried about endangering their health due to the nature of their work or work environment [68]. It encompasses both physical health anxiety and mental health anxiety. Severe health anxiety can cause many hazards, such as high levels of psychological stress, physical dysfunction, and excessive occupancy of healthcare facilities [69]. Health anxiety also affects individuals’ psychological functioning and increases employee turnover due to physical concerns, which affects employee productivity [70].

The risk of COVID-19 has emerged as a global public health issue [71]. During the COVID-19 pandemic, the number of hypochondriacs particularly prone to maladaptive behaviors has gradually increased. They excessively deny their emotions, which brings about severe health anxiety [72]. Health anxiety also adversely affects employee expectations, and increased job stress during COVID-19 can reduce employee performance [73]. 

The mining industry is a sector that accounts for many occupational injuries. Many miners suffer from respiratory occupational diseases, such as pneumoconiosis, asthma, and pulmonary edema as a result of long-term exposure to coal-mine dust. When miners are infected with COVID-19, the respiratory function of their lungs is directly affected, which threatens their lives and health. During COVID-19, workplace-safety risks for miners may impair many aspects, such as physical and mental health or job performance [74]. At the same time, occupational stress immensely influences employees’ health anxiety [75]. 

Undoubtedly, employees feel higher levels of job insecurity (i.e., through loss of income) during the COVID-19 pandemic, which seriously threatens their social status and performance [64]. Positive job insecurity and psychological stress can also lead to poor health [41]. In their study, Darvishmotevali et al. [76] observed that the performance of employees is affected by the job insecurity of hotel employees. 

During the COVID-19 pandemic, employees in many industries have experienced difficulties in achieving a work–family balance, in addition to the effects of job insecurity. For example, teachers have faced multiple layers of stress, which have gradually deteriorated their mental health and caused work–family conflicts. In addition, those in the healthcare industry cannot take on family responsibilities because of the increasing number of hours worked [77].

The substantial loss of resources (e.g., physical health, mental health, employment, financial status, and social relationships) caused by COVID-19 can easily create high levels of anxiety among employees [78]. Excessive occupational stress leaves workers prone to mental health problems and indirectly affects their job performance. Therefore, the following hypotheses were formulated:

**H6.**  *Health anxiety negatively mediates the relation between resource loss (perception of COVID-19 risks, life-safety risk, job insecurity, work–family conflicts) and job performance.*

**Figure 1:**
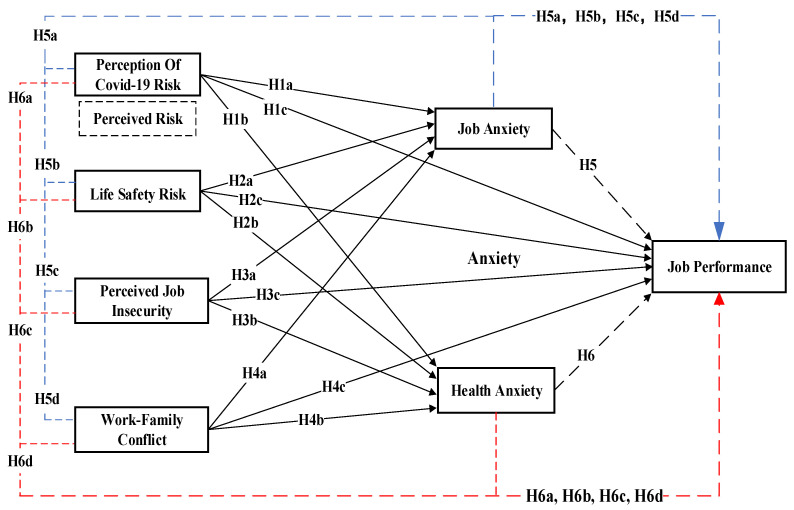
Conceptual framework. POCR—perception of COVID-19 risk; LSR—life-safety risk; PJI—perceived job insecurity; WFC—work–family conflict; JA—job anxiety; HA—health anxiety; JP—job performance.

Figure 1 shows the framework of the research model, which includes independent variables (perception of COVID-19 risks, life-safety risks, perceived job insecurity, and work–family conflicts), mediating variables (job anxiety and health anxiety), and dependent variables (job performance).

## 3. Methodology

### 3.1. Sample Selection and Data Collection

We used a multi-stage iterative process to collect the data. The subjects of this study were workers from a coal mine in China. Fifty questionnaires were presented to random respondents before the official release of the questionnaire. Second, respondents were interviewed to avoid problems such as vague expressions, unclear wording, and rhetorical errors in the questionnaire. Thirdly, in advance of the formal collection of the questionnaire, the original questionnaire was modified according to the previously collected information and respondents’ comments to guarantee the quality of the questionnaire. The official questionnaire was distributed to the workers in a coal mine in Huainan, China over 21 days, from 30 November to 20 December 2022, and a total of 715 questionnaires were collected. Excluding invalid questionnaires with consistent and incomplete answers, 629 valid questionnaires were recovered, with a valid response rate of 87.97%. From the collected data, the coal workers were all male, and the detailed demographic data are shown in Table 1.

### 3.2. Common Method Bias

This study addressed common method bias using Harman’s single-factor methodology. The first factor explained 40.097% of the variance, which is less than the 50% criterion [79], indicating that the common method bias in this study was not obvious.

### 3.3. Measures

The study questionnaire consisted of three general parts (e.g., study overview, questions related to demographic characteristics, questions related to variables). The previously developed and tested variable-item scales were used in this study. A five-point Likert scale was used for all seven variable-related measures, and five options ranging from 1 (“strongly disagree”) to 5 (“strongly agree”) were studied using a five-point Likert scale of strongly disagree, disagree, unsure, relatively agree, and strongly agree, respectively.

Perception of COVID-19 risk was assessed based on a questionnaire from a study by Yıldırım [80] with six measures. Sample items included “I am worried that I will accidentally get infected with COVID-19 and that it will cause a series of complications”: and “I am worried that there are significant sequelae to COVID-19 infection”.

Life-safety risks were assessed based on Hayes [81] research questionnaire with six measures Sample items included: “I feel that my life safety is affected by the dangerous and difficult working conditions in the underground workplace”; and “I feel that the intensity of work in the underground workplace affects my life safety”.

Perceived job insecurity was assessed based on a research questionnaire by Hellgren [39] and Witte [82] with four measures. Sample items included: “I am worried that I will be laid off if the mine needs to reduce production”; and “I believe that my current job at the mine is insecure”.

Work–family conflict was assessed based on a research questionnaire by Netemeyer [83] with seven measures. Sample items included: “Living apart makes it difficult for me to take care of my family responsibilities, causing work-family conflict”; and “The original family plan was broken, causing work–family conflict”.

Job anxiety was assessed based on De [62]’s research questionnaire with eight measures. Sample items included: “When I think about my work, I get pain in my chest”; and “I feel irritable or nervous because of my job”.

Health anxiety was assessed based on Jungmann [71]’s research questionnaire with six measures. Sample items included: “I find it difficult to think about other things when I notice that I am unwell”; “I have checked my body to check if there is something wrong with it”.

Job performance was measured with Teresa Aguiar-Quintana [64]’s research questionnaire, with six measures. Sample items included “The quality of my work is above the lowest standard for this job”; “I am more productive than others”.

### 3.4. Data Analysis

This study used SPSS 22.0 and Smart PLS software (version 3.3.9) to analyze the data. In addition, PLS-SEM was used to test hypotheses between the study variables (POCR, SLR, PJI, WFC, JA, HA, and JP) and to assess the plausibility of the measurement and structural models.

## 4. Results

### 4.1. Evaluation of Measurement Model

First, we consider the reliability test of the questionnaire. Typically, Cronbach’s alpha and composite reliability (CR) were used to evaluate the reliability of the model. The Cronbach’s alpha for all the measured variables in this measurement model was greater than the recommended value of 0.7, which ranged from 0.883 to 0.927. The CR varied from 0.919 to 0.941, which is higher than the commonly accepted criterion of 0.7, indicating the good reliability and internal consistency of the model variables.

The next measurement, validity, is usually conducted in two ways, i.e., convergent validity and discriminant validity. Convergent validity is a test that measures the consistency of multiple items for the same concept. Factor loadings and average variance (AVE) are valid indicators for testing convergent validity [84]. All 42 standardized factor loadings in the model are higher than 0.7 [85], which indicates that the model is highly correlated between the observed variables and the structural variables to which they belong. The AVE values vary between 0.657 and 0.739 with scores greater than 0.5 [86], indicating that the observed variables in the model explain each measurement dimension well. This analysis indicates that the evaluation structure of the measurement model is valid and reasonable. The reliability and convergent validity of each measure are shown in Table 2.

The discriminant validity was mainly verified by the square root of the AVE value (i.e., the diagonal value), and the remainder represented the correlation coefficient between the factors, which was greater than the correlation coefficient [87]. In addition, discriminant validity can also be determined by the results of the HTMT, which are usually lower than the accepted threshold of 0.85 [88]. Table 3 and Table 4 confirm the measurement of the discriminant validity.

Table 5 shows the factor-loading values for all the variables (bold values). A total of seven items were used to study the miners’ job performance, and all the items had values above 0.7.

Table 6 shows the variance influence factor (VIF) values for the independent variables (perception of COVID-19 risk, life-safety risk, perceived job insecurity, and work–family conflict), mediating variables (job anxiety and health anxiety), and dependent variable (job performance). All the VIF values were below the recommended threshold of 3.3 [89], indicating that our data analysis was not threatened by common method bias or multicollinearity.

Table 7 shows the fitness of the studied models. The standardized root-mean-square residual (SRMR) should be less than 0.08, according to Hu and Bentler [90]. The SRMR-estimated model value in this investigation was 0.042, while the saturated model’s value was 0.037. According to Bentler and Bonnet [91], the normed fit index (NFI) value ought to be higher than 0.80. The NFI saturated model score in this study was 0.902, and the NFI-estimated model value was 0.900. These numbers fall within the acceptable range.

### 4.2. Structural Model

**Figure 2:**
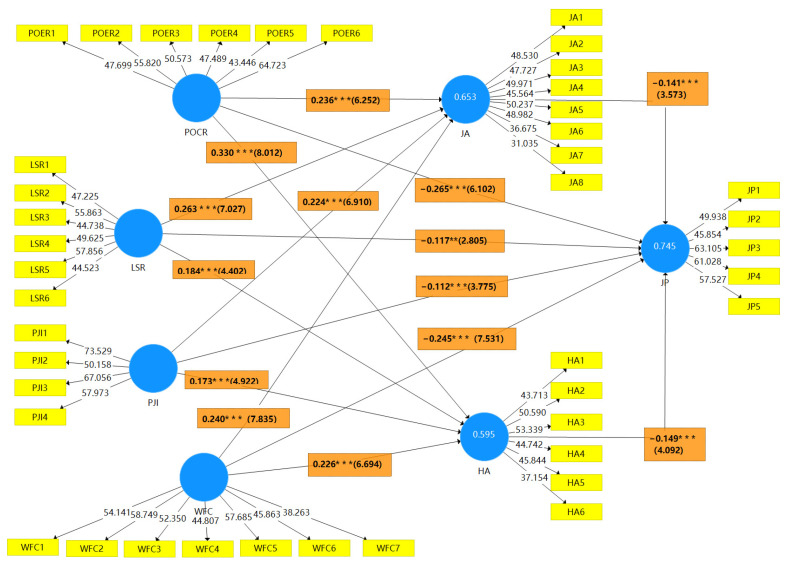
Graphical representation of the structural model. Note: ** *p* < 0.01, *** *p* < 0.001.

The structural model indicates the causal relationship between the variables in the model. It can visually reveal the internal relationships between potential variables. In the structural model, the estimated parameters show the direct influence of one structure on the other. Therefore, the significant coefficients at a certain level reveal the significant relationships between the potential structures [92]. In this study, the structural equations of the proposed theoretical model were analyzed using SmartPLS 3.0 software. Figure 2 shows the results of the analysis.

The results of the analysis of the above models showed that POCR (H1a, H1b) had a positive direct effect on JA and HA (β = 0.236, *p* < 0.001; β = 0.330, *p* < 0.001). However, H1c showed that POCR had a negative direct effect on JP (β = −0.265, *p* < 0.001). This implies that high POCR leads to low employee performance. The results of H2a and H2b show that LSR has a positive and significant direct effect on JA and HA (β = 0.263, *p* < 0.001; β = 0.184, *p* < 0.001). However, the results of H2c showed that LSR had a negative direct effect on JP (β = −0.117, *p* < 0.01). This implies that the higher the LSR, the lower the job performance. The results of H3a and H3b show that PJI has a positive direct effect on JA and HA (β = 0.224, *p* < 0.001; β = 0.173, *p* < 0. 001). Furthermore, H3c demonstrates that PJI has a negative effect on employees’ JP (β = −0.112, *p* < 0.001). These findings imply that high PJI results in poor employee job performance. The results of H4a and H4b show that WFC has a positive direct effect on JA and HA (β = 0.240, *p* < 0. 001; β = 0.226, *p* < 0.001), while H4c demonstrates that WFC negatively affects employees’ JP (β = −0.245, *p* < 0.001). This means that as the WFC increases, job performance decreases. It is assumed that H5 and H6 (JA and HA) have a significant negative effect on employees’ JP (β = −0.141, *p* < 0.001; β = −0.149, *p* < 0.001), as shown in Table 8 of the specific data results.

The indirect effects on the hypothesized results are shown in Table 9, where it is shown that JA mediated the relationship between POCR and JP (β = −0.033, *p* < 0.05; H5a). According to the findings of H6a, HA mediated the association between POCR and job performance (β = −0.049, *p* < 0.001). The results of H5b showed that JA mediated the relationship between LSR and JP (β = −0.037, *p* < 0.05). In addition, the results from H6b also showed that HA mediated the relationship between LSR and JP (β = −0.027, *p* < 0.05). According to the H5c results, JA mediated the relationship between PJI and JP (β = −0.032, *p* < 0.05). According to H6c, HA mediated the relationship between PJI and JP (β = −0.026, *p* < 0.05). In addition, JA mediated the relationship between WFC and JP (β = −0.049, *p* < 0.001; H5d) and HA mediated the relationship between WFC and JP (β = −0.034, *p* < 0.001; H6d). All the hypothesized direct and indirect effects were significant and partially mediated by JA and HA.

Table 10 shows the values of the variables R^2^ and Q^2^. The value of R^2^ indicates the proportion of variance in the dependent variable explained by its predictor variables. The adjusted R^2^ values for job performance were 0.745, JA (0.651) and HA (0.593). Overall, the R^2^ values were found to be consistent with the recommended critical value of 0.33 [93]. The Q^2^ values for job anxiety, health anxiety, and job performance were 0.424, 0.390, and 0.540, respectively, with larger Q^2^ values indicating stronger predictive correlations [94].

## 5. Discussion

As essential members of the coal industry, miners play an irreplaceable role in coal production. Therefore, the production performance of miners has a direct impact on the development of the energy economy. From 2020 to the time of writing, the impact of COVID-19 has not disappeared. During this period, the coal-mining industry has suffered various losses of resources (e.g., health, working conditions, finances, and family). These losses have exacerbated miners’ psychological conditions, which has affected the performance of these employees. To explain the impact of resource loss on employee performance, this discussion provides an in-depth analysis of the current results based on the previous literature. This study examines miners’ anxiety and job performance from a resource-loss perspective. It ultimately provides an understanding of the impact of COVID-19-related emergencies on the job performance of Chinese miners.

First, the perception of COVID-19 risk has positively and significantly affected job and health anxiety during the COVID-19 pandemic (H1a, H1b). In the early stage of the full liberalization of epidemic-prevention-and-control measures, the rate of COVID-19 infection among Chinese people rapidly increased in a short period of time. The Chinese government ceased the adoption tight measures to restrict the spread of the virus. Thus, many previously uninfected miners experienced increased fear of COVID-19. This outcome is in line with those of previous studies, which demonstrated that the high contagiousness of COVID-19 makes miners fear being infected [29]. The severe impact of COVID-19 creates a convergence of anxiety levels between individuals. Some related studies reported symptoms of anxiety in healthcare workers during the COVID-19 pandemic [77]. Sarfraz et al. indicated that health anxiety associated with COVID-19 led to poorer job performance among healthcare workers [70]. These studies support our results and H1c. Previous studies also found that the occupational risks to which miners are exposed can cause psychological problems and job anxiety [95]. Harsh underground working environments and hypertension may be the main factors in miners’ health anxiety [96]. In addition, some personal characteristics (such as occupational and equipment factors) that cause life-safety risks significantly affect the psychological health of employees through effects such as job and health anxiety [21]. For miners, occupational risk hinders job performance and increases psychological problems, such as anxiety [96]. Problems related to work execution, such as dangerous working environments and high job complexity, continuously plague miners’ job performance [97]. Therefore, these research findings support H2a, H2b, and H2c. Miners were always at high risk of infection through coal mining, regardless of whether their employers adopted a closed-management or a liberalized policy. If older miners have underlying diseases, their lives may be at risk, reducing their productivity. 

The results also showed that perceived job insecurity positively affects job anxiety and health anxiety (H3a, H3b). These findings are in line with those of previous studies, which found that the economic losses due to the COVID-19 pandemic have created the idea that miners “could lose their jobs at any time”, an idea that continues to torment them physically and mentally [54]. Employees are vulnerable to anxiety when faced with economic instability and job insecurity [98]. Similarly, the shock of job instability generates psychological problems, which can decrease employee productivity [39]. The results of these studies support H3c. When the Chinese government abandoned its COVID-19-related emergency policy early in the pandemic, increasing numbers of people were absent from work and could not return to work because of the pandemic. These factors made it impossible for employees to obtain job security.

The working population in China has faced severe work–family-conflict problems, especially in the coal industry [53]. Previous studies showed that work–family conflict not only increases psychological barriers for miners, but may also contribute to coal-production accidents [99]. Miners’ average workload and work hours have increased, creating a positive association between work–family conflict and anxiety symptoms during the COVID-19 pandemic [55]. Work–family conflict can distract miners and lower job performance [19]. These results support H4a, H4b, and H4c. COVID-19 has exacerbated the work-family conflict of miners. Many coal mines have a closed management policy whereby miners live and work in the mines, which leads to them being separated from their families. Some miners could not return to work because of home quarantine. These factors have caused work–family conflicts for miners. 

The loss of resources due to COVID-19 (i.e., the perception of COVID-19 risk, life-safety risk, job insecurity, and work–family conflicts) significantly affects employee performance. Some studies have shown that the job uncertainty due to COVID-19 exacerbates occupational panic [98]. The specific occupational nature of mining may also aggravate job anxiety and health anxiety and hinder miners’ ability to perform. In addition, job insecurity can increase anxiety and negatively affect miners’ performance during the COVID-19 pandemic [100]. This increasing risk, job insecurity, and family–work conflicts are closely related to anxiety and stress, ultimately hindering employee performance. The miners’ poor work status and health can negatively affect their performance (H5, H6). To summarize, this study’s results support the acceptance of all the proposed hypotheses.

In conclusion, this study built a model based on different dimensions of resource loss and miners’ job performance based on the resource-conservation theory. We investigated the roles of job anxiety and health anxiety as mediators of four aspects of resource loss that indirectly affect job performance: the perception of COVID-19 risk (the health dimension); the perception of life-safety risk (the occupational dimension), perceived job insecurity (the financial dimension), and work–family conflict (the family dimension). Our study offers several theoretical contributions. First, this study expands the literature on Chinese miners. Second, it adds miners as a study group to the field of research on COVID-19. Third, the consequences of the epidemic were still evident despite the fact that the survey was conducted around the time when China’s COVID-19 emergency program was liberalized. Therefore, the context of this study of miners’ anxiety and job performance is unique. Fourth, The COR theory’s range of applications was increased. In a public-crisis event (i.e., COVID-19), miners’ anxiety and job performance were examined from a resource-loss perspective.

This study can provide valuable information to stakeholders in the coal-production industry and other policy researchers about the anxiety produced by public-health emergencies among miners. Regarding occupational safety, coal-mine managers should prepare for epidemic prevention, control, and emergency response to ensure occupational health and safety in emergencies (such as the COVID-19 crisis). For example, coal companies should regularly disinfect and clean their workplaces to ensure a safe working environment during the COVID-19 pandemic. Managers should promptly seal off potentially infected environments and isolate suspected patients for observation to reduce the risk of COVID-19 infection. Second, regarding job security, coal companies should increase internal communication and release official information about employment policies or security through social networks and the internet. Employees should be treated appropriately during work stoppages, in accordance with laws and regulations. Third, regarding work and family, managers should focus on miners’ families, support work–family-boundary management, and create an awareness of work–family boundaries. For example, the rationalization of workloads and communication time with family members should be increased for returning miners. For non-returning workers, management should improve communication with miners. Fourth, coal companies need to pay attention to the psychological problems of employees and provide psychological counseling and communication to miners in a timely manner by setting up psychological consultation rooms. Psychologists and sociologists should regularly be invited to the mines to conduct in-depth investigations and studies. In the face of crisis events, managers should provide sufficient resources to prevent their employees’ performance from being affected.

This study also has several limitations. First, this paper used cross-sectional data, but future studies might take longitudinal data into account. Second, we collected data from only one coal mine in Huainan, China, and the sample size may have limited the generalizability of the model. In addition, miners are a relatively large group, but our sample size was small. Therefore, we expanded the sample scope and size later in the study. Third, the dependent variable in our study model was resource loss, and no interactions between the dependent variables were considered. Furthermore, the variable design did not include moderating variables to study the miners’ job performance. Therefore, in future studies, we will consider the interaction of resource losses and include some moderating variables when analyzing miners’ job performance. Fourth, the questionnaire was administered before and after the abandonment of the COVID-19-emergency policy in China. Although the effects of the COVID-19 pandemic did not disappear, the subjective attitudes of miners changed over time, along with the guidelines. 

## 6. Conclusions

The severe effects of COVID-19 have affected coal miners’ occupational lives. The high contagiousness of COVID-19 has seriously hampered the mental health of employees and their productivity at work. This makes frontline workers vulnerable and generates various resource losses, in addition to health risks.

This paper investigated the severe impact of the COVID-19 pandemic on the Chinese coal-mining industry. The findings support a negative relationship between resource loss (health, work environment, finances, and family) and work performance. The increased job and health anxiety among the miners made them unable to perform their jobs and negatively affected productivity. Similarly, the results showed that job performance was strongly influenced by COVID-19-related stress. The impact of the COVID-19 pandemic also showed how employee anxiety and job performance were affected by COVID-19 risk, life-safety risk, job uncertainty, and family–work conflict. In addition, the study showed that work and health anxiety had a substantial mediating role in influencing employee performance.

Undoubtedly, COVID-19 has created a severe challenge for the coal-mining industry. The impact of COVID-19 has drawn the attention of researchers to the mental health (i.e., anxiety) of individuals. The results of this study indicate that coal managers monitor the mental health of their workers. They should implement strategies that include educating people about mental health management, as well as the effective deployment of management techniques to reduce anxiety in the workplace and improve employee performance. In conclusion, policymakers and coal-mine practitioners should take emergency management measures to improve employee performance and mental health in the event of a crisis event.

## Figures and Tables

**Table 1:** Study participants’ demographic information.

Items		Frequency (N = 629)	(%)
Educational background	Middle school and below	194	30.84
	High school	182	28.93
	College	141	22.42
	Undergraduate	82	13.04
	Master’s degree and above	30	4.77
Age	≤25	167	26.55
	26–35	159	25.28
	36–45	105	16.69
	≥46	198	31.48
Working years	≤5	88	13.99
	5–10	167	26.55
	11–20	187	29.73
	21–30	120	19.08
	≥30	67	10.65
Marital Status	Single	535	85.1
	Married	94	14.9
Monthly income	≤2000	45	7.15
	2001–4000	51	8.11
	4001–6000	141	22.41
	6001–8000	229	36.41
	8001–10,000	130	20.67
	≥10,000	33	5.25

**Table 2:** Reliability and validity tests.

Construct	Items	Loading	α	CR	AVE
POCR	POCR1	0.819	0.923	0.940	0.722
	POCR2	0.841			
	POCR3	0.822			
	POCR4	0.838			
	POCR5	0.842			
	POCR6	0.817			
LSR	LSR1	0.820	0.909	0.929	0.687
	LSR2	0.841			
	LSR3	0.822			
	LSR4	0.838			
	LSR5	0.842			
	LSR6	0.816			
PJI	PJI1	0.867	0.883	0.919	0.739
	PJI2	0.845			
	PJI3	0.866			
	PJI4	0.862			
WFC	WFC1	0.835	0.927	0.941	0.697
	WFC2	0.853			
	WFC3	0.855			
	WFC4	0.834			
	WFC5	0.847			
	WFC6	0.825			
	WFC7	0.803			
JA	JA1	0.824	0.925	0.939	0.657
	JA2	0.815			
	JA3	0.832			
	JA4	0.810			
	JA5	0.829			
	JA6	0.829			
	JA7	0.784			
	JA8	0.762			
HA	HA1	0.812	0.899	0.923	0.665
	HA2	0.831			
	HA3	0.839			
	HA4	0.824			
	HA5	0.814			
	HA6	0.784			
JP	JP1	0.849	0.910	0.933	0.735
	JP2	0.835			
	JP3	0.878			
	JP4	0.864			
	JP5	0.861			

**Table 3:** Discriminant-validity analysis (Fornel–Larcker).

Constructs	AVE	HA	JA	JP	LSR	PJI	POCR	WFC
HA	0.665	0.816						
JA	0.657	0.751	0.811					
JP	0.735	−0.734	−0.751	0.857				
LSR	0.687	0.644	0.697	−0.696	0.829			
PJI	0.739	0.626	0.674	−0.678	0.629	0.860		
POCR	0.722	0.699	0.701	−0.761	0.696	0.643	0.850	
WFC	0.697	0.609	0.640	−0.701	0.535	0.550	0.573	0.835

**Table 4:** Discriminant-validity analysis (HTMT).

Constructs	HA	JA	JP	LSR	PJI	POCR	WFC
HA							
JA	0.821						
JP	0.811	0.818					
LSR	0.708	0.756	0.763				
PJI	0.700	0.744	0.756	0.699			
POCR	0.765	0.755	0.829	0.755	0.710		
WFC	0.665	0.688	0.763	0.580	0.606	0.619	

**Table 5:** Discriminant-validity analysis (cross-loadings).

Construct’s Items	HA	JA	JP	LSR	PJI	POCR	WFC
HA1	**0.810**	0.621	−0.588	0.530	0.500	0.551	0.482
HA2	**0.829**	0.582	−0.589	0.485	0.493	0.523	0.466
HA3	**0.836**	0.639	−0.618	0.554	0.537	0.612	0.516
HA4	**0.821**	0.607	−0.592	0.521	0.514	0.580	0.481
HA5	**0.814**	0.620	−0.615	0.525	0.534	0.581	0.526
HA6	**0.784**	0.604	−0.586	0.532	0.481	0.570	0.507
JA1	0.622	**0.822**	−0.605	0.584	0.563	0.569	0.524
JA2	0.645	**0.816**	−0.634	0.579	0.546	0.596	0.548
JA3	0.608	**0.832**	−0.619	0.561	0.567	0.576	0.540
JA4	0.614	**0.812**	−0.619	0.567	0.553	0.592	0.532
JA5	0.644	**0.829**	−0.616	0.587	0.573	0.605	0.521
JA6	0.649	**0.827**	−0.642	0.598	0.554	0.613	0.539
JA7	0.548	**0.782**	−0.586	0.531	0.491	0.506	0.487
JA8	0.529	**0.762**	−0.545	0.505	0.518	0.472	0.449
JP1	−0.617	−0.646	**0.849**	−0.612	−0.570	−0.665	−0.621
JP2	−0.631	−0.638	**0.835**	−0.580	−0.563	−0.633	−0.578
JP3	−0.638	−0.664	**0.878**	−0.590	−0.607	−0.651	−0.599
JP4	−0.636	−0.651	**0.864**	−0.630	−0.579	−0.677	−0.620
JP5	−0.623	−0.621	**0.861**	−0.569	−0.587	−0.633	−0.586
LSR1	0.561	0.592	−0.601	**0.822**	0.539	0.622	0.465
LSR2	0.546	0.586	−0.574	**0.839**	0.504	0.579	0.448
LSR3	0.549	0.576	−0.581	**0.819**	0.528	0.586	0.438
LSR4	0.516	0.576	−0.557	**0.837**	0.530	0.547	0.434
LSR5	0.567	0.622	−0.619	**0.841**	0.550	0.616	0.476
LSR6	0.449	0.502	−0.520	**0.815**	0.473	0.496	0.392
PJI1	0.583	0.611	−0.615	0.614	**0.866**	0.595	0.517
PJI2	0.500	0.536	−0.572	0.498	**0.845**	0.516	0.447
PJI3	0.526	0.574	−0.566	0.556	**0.868**	0.559	0.450
PJI4	0.538	0.592	−0.577	0.490	**0.861**	0.539	0.473
POCR1	0.588	0.589	−0.621	0.599	0.543	**0.854**	0.469
POCR2	0.603	0.608	−0.655	0.586	0.546	**0.858**	0.487
POCR3	0.620	0.608	−0.664	0.608	0.566	**0.844**	0.498
POCR4	0.602	0.618	−0.663	0.614	0.557	**0.829**	0.511
POCR5	0.577	0.580	−0.652	0.584	0.547	**0.834**	0.472
POCR6	0.570	0.566	−0.621	0.553	0.516	**0.879**	0.483
WFC1	0.519	0.556	−0.606	0.456	0.467	0.499	**0.837**
WFC2	0.513	0.536	−0.579	0.455	0.471	0.490	**0.850**
WFC3	0.520	0.549	−0.598	0.464	0.468	0.458	**0.855**
WFC4	0.516	0.560	−0.589	0.468	0.462	0.489	**0.833**
WFC5	0.531	0.556	−0.597	0.463	0.480	0.504	**0.845**
WFC6	0.483	0.502	−0.580	0.420	0.440	0.467	**0.822**
WFC7	0.472	0.474	−0.547	0.396	0.423	0.442	**0.801**

Note: bolded values—factor loading; non-bolded values—cross-loading.

**Table 6:** Variance influence factor.

Constructs	HA	JA	JP	LSR	PJI	POCR	WFC
HA			2.81				
JA			3.282				
JP							
LSR	2.227	2.227	2.446				
PJI	2.023	2.023	2.19				
POCR	2.379	2.379	2.703				
WFC	1.663	1.663	1.881				

**Table 7:** Model Fit.

	Fit Indexes	Saturated Model	Estimated Model
SRMR	<0.08	0.037	0.042
NFI	>0.80	0.902	0.900
Chi-square		2151.481	2212.82

**Table 8:** Hypotheses testing—direct effects.

Hypothesis	Direct Relationships	Std. Beta	Std. Error	T Values	*p* Values
H1a	POCR→JA	0.236	0.034	6.892	***
H1b	POCR→HA	0.330	0.039	8.541	***
H1c	POCR→JP	−0.265	0.041	6.411	***
H2a	LSR→JA	0.263	0.036	7.209	***
H2b	LSR→HA	0.184	0.039	4.727	***
H2c	LSR→JP	−0.117	0.039	2.968	**
H3a	PJI→JA	0.224	0.033	6.820	***
H3b	PJI→HA	0.173	0.036	4.783	***
H3c	PJI→JP	−0.112	0.028	3.934	***
H4a	WFC→JA	0.240	0.033	7.293	***
H4b	WFC→HA	0.226	0.034	6.719	***
H4c	WFC→JP	−0.245	0.033	7.409	***
H5	JA→JP	−0.141	0.040	3.547	***
H6	HA→JP	−0.149	0.035	4.243	***

Note: ** *p* < 0.01, *** *p* < 0.001.

**Table 9:** Hypothesis results—indirect effects.

Hypothesis	Indirect Relationships	Std. Beta	Std. Error	T Values	*p* Values
H5a	POCR→JA→JP	−0.033	0.011	3.106	**
H5b	LSR→JA→JP	−0.037	0.012	3.163	**
H5c	PJI→JA→JP	−0.032	0.010	3.160	**
H5d	WFC→JA→JP	−0.034	0.011	3.158	**
H6a	POCR→HA→JP	−0.049	0.013	3.847	***
H6b	LSR→HA→JP	−0.027	0.009	3.001	**
H6c	PJI→HA→JP	−0.026	0.009	3.009	**
H6d	WFC→HA→JP	−0.034	0.009	3.683	***

Note: ** *p* < 0.01, *** *p* < 0.001.

**Table 10:** Quality criteria (R^2^ and Q^2^).

Latent Variables	R^2^	R^2^ Adjusted	Q^2^
HA	0.595	0.593	0.390
JA	0.653	0.651	0.424
JP	0.745	0.743	0.540

## Data Availability

The current study data can be obtained from the corresponding author.
